# Soy Metabolism by Gut Microbiota from Patients with Precancerous Intestinal Lesions

**DOI:** 10.3390/microorganisms8040469

**Published:** 2020-03-25

**Authors:** Lorenzo Polimeno, Michele Barone, Adriana Mosca, Maria Teresa Viggiani, Farahnaz Joukar, Fariborz Mansour-Ghanaei, Sara Mavaddati, Antonella Daniele, Lucantonio Debellis, Massimo Bilancia, Luigi Santacroce, Alfredo Di Leo

**Affiliations:** 1 Polypheno Academic Spin Off, University of Bari “A. Moro”, 70124 Bari, Italy; lorenzo.polimeno@uniba.it; 2 Gastroenterology Unit, Department of Emergency and Organ Transplantation, University of Bari, 70124 Bari, Italy; michele.barone@uniba.it (M.B.); mariateresa.viggiani@uniba.it (M.T.V.); alfredo.dileo@uniba.it (A.D.L.); 3 Interdisciplinary Department of Medicine (DIM), University of Bari “Aldo Moro”, Policlinico, Piazza G. Cesare 11, 70124 Bari, Italy; adriana.mosca@uniba.it; 4 Gastrointestinal and Liver Diseases Research Center, Guilan University of Medical Sciences, 41448-95655 Rasht, Iran; farajov@gmail.com (F.J.); ghanaie@yahoo.com (F.M.-G.); s.maveddati@alumni.ut.ac.ir (S.M.); 5 Experimental Oncology, Scientific Institute for Cancer Care and Research IRCCS “G. Paolo II”, Viale Orazio Flacco, 65, 70124 Bari, Italy; anto.dani27@gmail.com; 6 Department of Biosciences, Biotechnologies and Biopharmaceuticals, University of Bari “Aldo Moro”, Via E. Orabona 4, 70124 Bari, Italy; lucantonio.debellis@uniba.it; 7 Ionian Department (DJSGEM), University of Bari “Aldo Moro”, 74123 Taranto, Italy; massimo.bilancia@uniba.it; 8 Microbiology and Virology Lab., Policlinico University Hospital of Bari, 70124 Bari, Italy

**Keywords:** anaerobic bacteria, gut microbiota, intestinal polyps, metabolomics, soy, phytoestrogen, equol, daidzein

## Abstract

Background: Colorectal cancer (CRC) requires the presence of a variety of factors predisposing a tumorigenic milieu. Excluding familial clustering and hereditary CRC syndromes, the development of sporadic CRC from precancerous lesions is influenced by tissue inflammation, modulation of intestinal immunity, hormones, dietary habits and gut microbiota composition. As concerning the last two aspects, the intestinal presence of equol, the most biologically active metabolite of the soy isoflavone daidzein and the presence of a genetic determinant of gut microbiota able to metabolize daidzein, seem to lower the CRC risk. It has been hypothesized that the anaerobic microorganisms of the *Bacteroides* genus play a role in equol production. Aim: To evaluate the presence of (i) anaerobic gut microbiota and (ii) the urinary levels of soy isoflavones (daidzein, genistein and equol) in patients with and without precancerous lesions, challenged with a daidzein-rich soy extract. Methods: Consecutive subjects undergoing colonoscopy participated to the study. Feces were collected from all patients one week before colonoscopy for gut microbiota studies. After the endoscopy examination and the histological evaluation, 40 subjects, 20 with sporadic colorectal adenomas (SCA/P group) and 20 without proliferative lesions (control group) were enrolled for the study. Urine levels of soy isoflavones daidzein, genistein and their metabolite equol, were determined by high performance liquid chromatographic (HPLC) analysis and gut microbiota analysis was performed by Matrix Assisted Laser Desorption Ionization Time of Flight Mass Spectrometry (MALDI-TOF MS) procedure. Results: Seventeen different bacterial species were identified in the fecal samples of the forty subjects participating to the study. Ten bacterial species resulted anaerobic Gram-negative bacteria, all belonging to the *Bacteroides* genus. A significant difference of bacteria species was evidenced in the fecal samples of the two groups of subjects. Particularly important was the evidence of *Parabacteroides distasonis, Clostridium clostridioforme* and *Pediococcus pentasaceus* only in control fecal samples, such as the presence of *Bacteroides fragilis* and *Prevotella melaningenica* only in SCA/P fecal samples. Concerning the soy isoflavones levels, no statistically significant differences were revealed in the genistein and daidzein urinary levels between the two groups of subjects. On the contrary, urinary equol levels were undetectable in ten SCA/P subjects and in two controls; moreover, when present, the levels of urinary equol were significantly lower in SCA/P subjects compared to controls (0.24 ± 0.27 mg/24 hrs *vs*. 21.25 ± 4.3 mg/24 hrs, respectively, *p* = 1.12 × 10^−6^). Conclusions: Our results suggest that the presence of anaerobic *Bacteroides* in the colon, and the production of equol from soy, could determine a milieu able to contrast the development of colonic mucosa proliferative lesions.

## 1. Introduction

Colorectal cancer (CRC) is the third most prevalent cancer and one of the most common solid carcinomas in Western countries. The worldwide incidence rate of CRC widely varies, resulting higher in more industrialized countries than in developing countries, being 10-fold higher in the United States and Europe than in African and Asian countries [1]. Western lifestyle, dietary habits (red meat, alcohol consumption, low fibers intake, etc.) and obesity represent important risk factors for CRC development [2]. The development of CRC requires also genetic, inflammatory and immunological factors and is influenced by sex hormones and, just as important, the presence of a genetic determinant of gut microbiota. The metabolomic exposure to all these factors can be pro- or anti-carcinogenic in nature and sometimes can enable and model a tumorigenic milieu [3,4,5,6,7,8,9]. 

Having this in mind, we are convinced that modern lifestyle, and specifically the nutritional habits, which can alter gut microbiota, can influence colon cancer development and, acting as pro-carcinogenic, promote inflammation [10]. In fact, a high protein intake, increasing branched-chain fatty acids and phenylacetic acid production that, once metabolized by gut microbiota, produces phenolic compounds, indoles and *p-cresol*, proved to be responsible for CRC induction [11,12,13,14]; in addition, diets rich in saturated fats increase the secondary bile acids synthesis in CRC patients compared to healthy controls [15]. On the contrary, beneficial aspect, for the colon health, is covered by a rich-fibers diet (i.e., Mediterranean diet) and by the presence of “good” intestinal bacteria, such as the *Bacteroidetes* and *Firmicutes* phyla, able to produce short chain fatty acids (SCFAs), generally considered to reduce CRC risk [16] and reported dramatically reduced in the gut of tumour-bearing mice; intake of fruits, vegetables and legumes have been reported to decrease the risk for colorectal adenomas and CRC and, positively, regulate gut microbiota [17,18]. 

It is important to underline that the majority of gut microbiota (i) is not pathogenic and plays a symbiotic role; (ii) has a crucial protective function against pathogenic bacteria colonization and growth, consumes nutrients and/or produces bacteriocins/antimicrobial peptides, modifies intestinal pH and affects cell signalling pathways; (iii) prevents invasion of pathogenic bacteria and protects the intestinal permeability [19]. *Parabacteroides distasonis* (*Pa.d.*) is a “good” bacterial genus known to exert anti-inflammatory and anti-cancer properties [20], lowering intestinal inflammation and its pathological consequences [21].

As a confirmation of what has been reported about the influence of diet on cancer incidence, if ever necessary, strong epidemiological studies report significant differences in CRC incidence among ethnic groups having different nutritional habits, which, in turn, can modulate gut microbiota composition [22,23]. Lower CRC incidences in Far Eastern countries has partly been attributed to high nutritional intake of soy and its abundance in isoflavones. Isoflavones are plant-derived substances which, due to molecular similarities to endogenous estrogens, act as phytoestrogens interacting with estrogen receptors [3,4,5]. The induction of apoptosis and inhibition of tyrosine kinases and DNA topoisomerases have been shown to be responsible for possible anti-carcinogenic properties of phytoestrogens [24,25,26]. Among the soy metabolites, of interest is the availability of daidzein and its most biologically active metabolite, equol, produced only by gut microbiota intervention [27]. Equol, exerting anti-carcinogenic and antioxidative properties, has been reported to be inversely associated with CRC risk [28]. The isoflavones can be hydrolyzed along the entire length of the gastrointestinal tract, but mostly, they are hydrolyzed in the jejunum and, by the cooperation of the brush border membrane and bacterial β-glucosidases, are subsequently absorbed, as aglycons, across the intestinal epithelium. Part of the isoflavones passes to the large intestine, where the glycosylated, sulphated and glycuronicated forms are deconjugated by bacterial enzymes and subsequently absorbed or further metabolized by intestinal microflora. Daidzein is metabolized to dihydrodaidzein, which is further converted to equol or O-desmethylangolensin (O-DMA) [29].

We recently reported a loss of intestinal anaerobic bacterial diversity in subjects with sporadic colorectal adenomas, of *Bacteroides* genus in particular [30]. With the present project, we studied the intestinal microenvironment (gut microbiota and soy metabolism) with the intent to establish if it could contribute to the formation of a predisposing carcinogenetic milieu. For this reason, we challenged with a food supplement based on standardized soy extract in daidzein-rich isoflavones, subjects who performed colonoscopy and showed either sporadic colorectal adenomas (SCA/P) or no proliferative lesions (control group), to evaluate urinary levels of soy isoflavones, and fecal gut microbiota. 

## 2. Materials and Methods 

### 2.1. Patients Enrollment

Consecutive subjects undergoing colonoscopy at the Gastroenterology Unit, Policlinico University Hospital, Bari, Italy, participated to the study. Feces were collected from all subjects before colonoscopy for gut microbiota studies. After endoscopy examination and histological evaluation, 40 subjects, 20 with sporadic colorectal adenomas (SCA/P group) and 20 without proliferative lesions (control group), matched for age and sex, were enrolled. The study was conducted in agreement with the ethical guidelines of the Declaration of Helsinki and received the approval by the local Ethics Committee of the University of Bari (Prot.CE/4748, 29 July 2016) and was registered on www.ClinicalTrials.gov (# NCT03417258). 

The inclusion criteria were subjects of both sexes aged between 50 and 75 years, undergoing colonoscopy. This age range was adopted for several reasons: screening colonoscopies are prescribed specially in subjects over 50 years, when the probability to find neo-proliferative lesions is increased; patients with a family history of CRC frequently develop polyps at a younger age; elderly patients may have had trouble continuing the study. The exclusion criteria were: age < 50 and > 75 years, previous diagnosis of colon cancer or inflammatory bowel diseases (IBD), hereditary intestinal tumours, ongoing infections, intake of pre- or probiotics and synbiotics, or drugs (e.g., antibiotics) that could alter the intestinal microbiota during the 4 weeks that preceded colonoscopy, creatinine clearance below 60 mL/min, decompensated liver.

**Scheme 1:**
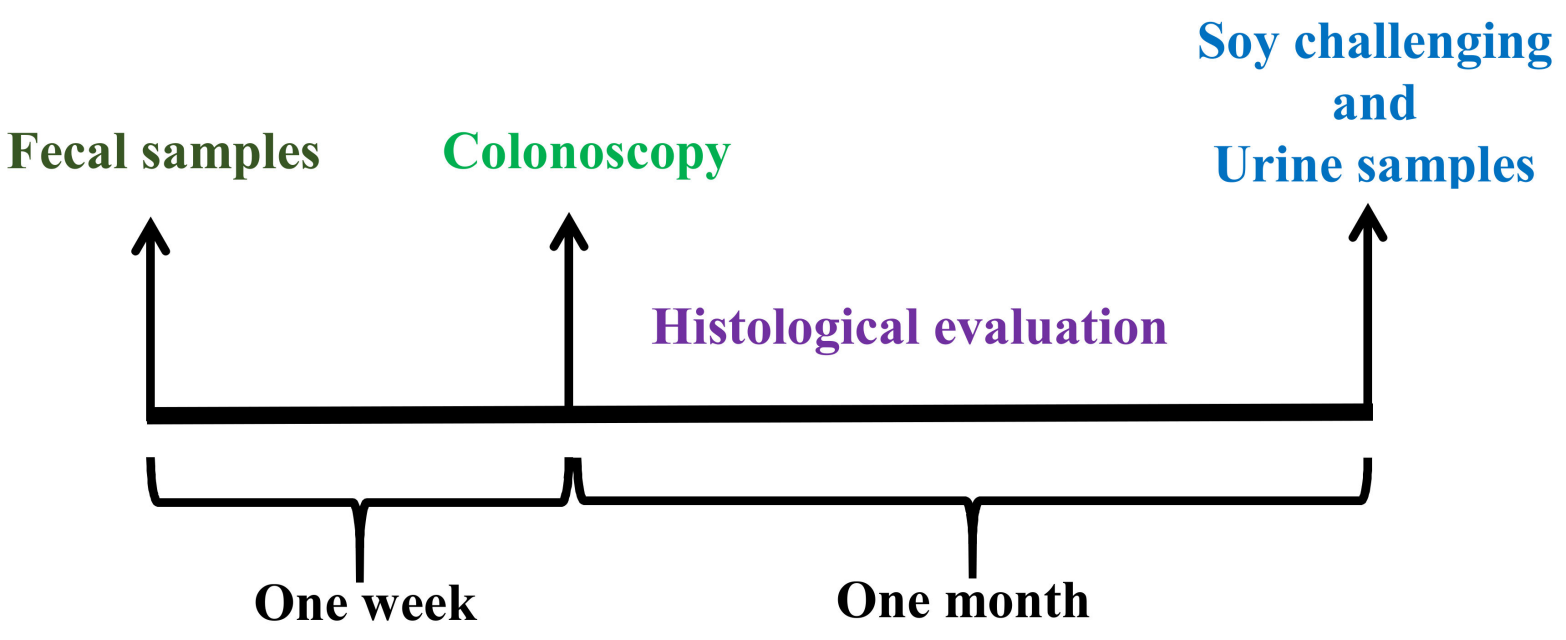
The study protocol timetable.

The following determinations were performed on the biological samples of all subjects: (i) anaerobic gut microbiota evaluation in fecal samples by MALDI-TOF MS analysis, (ii) isoflavonoids assessment (daidzein, genistein and equol) in urine samples by HPLC. Gut microbiota analysis was performed on fecal samples obtained one week before colonoscopy, while isoflavonoid urinary excretion was determined 1 month after colonoscopy (after the histological evaluations were performed), as reported in the following Scheme 1 of protocol study:

The demographical and anthropometric parameters of the patients involved in the study, matched for sex, age and BMI, are reported in Table 1. No statistical difference was detected between the two groups of subjects in the reported parameters.

### 2.2. Microbiological Study

Fecal samples were collected from each subject and immediately analyzed for the presence of the anaerobic bacteria. The stool samples were inoculated on both Schaedler blood agar and on Bile Esculin Agar (BEA) as selective isolation medium for anaerobic bacteria *Bacteroides* spp. After incubation at 37 °C for 72 h in an anaerobic jar, strict anaerobes were chosen. Thereafter, after grown on both culture plates, identification of the different colonies was performed by MALDI-TOF MS analysis, an innovative technology for the rapid and accurate identification of bacterial and fungal isolates in clinical settings [31]. MALDI-TOF MS technology has been developed to profile, within minutes instead of the usually required 36–48 h of the traditional approaches, based on the pure culture of the microorganism, bacterial proteins from whole cell extracts and to obtain a bacterial fingerprint able to discriminate microorganisms from different genera and species [32]. The technology is automated, high throughput, and applicable to a broad range of common as well as esoteric bacteria and fungi.

### 2.3. High Performance Liquid Chromatographic Assay of Isoflavonoids

For human urine analysis of the most common dietary isoflavones, daidzein, genistein and their mammalian metabolite equol, we applied the HPLC assay described by Franke [33], with minor revisions as described in detail below.

#### 2.3.1. Instrument

HPLC analysis were carried out on a System chromatograph LC20 Chromatography Enclosure (Thermo Fisher Scientific Inc., Monza, Italy), with a PDA-100 Photodiode Array Detector and a Fluorescent detector Ultimate 3000.

#### 2.3.2. Chemicals

Anhydrous powders (sodium acetate, sodium ascorbate, boric acid, ascorbic acid) and all the chemical reagents were purchased, if not specifically reported, from Sigma-Aldrich (Milan, Italy). Distilled water, 96% ethanol, dimethyl-sulfoxide (DMSO), acetonitrile, 99.8% glacial acetic acid, 100% methanol and all solvents used for HPLC and optical density readings were analytical grade or HPLC grade from Fisher Scientific (Monza, Italy). Crystalline standards, Daidzein (DE), Genistein (GE), Equol (EQ), Flavone were also purchased from Fisher Scientific (Monza, Italy). β-Glucuronidase/Arylsulfatase (isolated from *Helix pomatia* type HP-2S) was purchased from Sigma-Aldrich (Milan, Italy).

#### 2.3.3. Standard Solutions and Calibration Curves 

Phytoestrogen stock solutions (daidzein, genistein and equol) and flavone were prepared dissolving the powder standards in 5.0 mL of DMSO, then adding 96% ethanol to reach 10 mM solutions. Each stock solution was then diluted with 96% ethanol to obtain the calibration curves with the following concentration values for each standard: daidzein 0.5 mM, 0.25 mM and 0.16 mM; genistein 0.50 mM, 0.25 mM and 0.12 mM; equol 1 mM, 0.50 mM and 0.25 mM; flavone 2 mM, 1 mM and 0.50 mM.

#### 2.3.4. Chromatographic Study

Reversed phase columns *Hypersep C-18* supplied by Thermo Scientific were used to carrier out a solid phase extraction (SPE). HPLC analysis was carried out using a PolarAdvantage II (4.6 × 150 mm; 3 μm) C18 column directly connected to an *Adsorbosphere C18* precolumn. The injection volume of analysis samples was of 100 μL. Elution was carried out at a flow rate of 0.80 mL/min for 40 min using, as mobile phase, a mixture of water and acetonitrile (A = acetonitrile, B = distillated water) characterized by the following gradient: 20% A in B for 16 min, from 16 to 30 min the mixture gradually changes to 70% A, remaining stable for 5 min, then returning to 20% A until the end of the analysis. Analytes were monitored with a diode array detector using 260 nm wavelengths for daidzein, genistein and flavone detection, and 280 nm for equol detection. CHROMELEON software connected to the chromatographing system was used for the acquisition and processing of data. 

#### 2.3.5. Soy Challenging and Urine Collection

Urinary levels of phytoestrogens after soy derived product challenge were evaluated in each subject keeping separately the first and second 12 h urine collection. All enrolled subjects received 4 capsules of a food supplement based on standardized soy extract in daidzein-rich isoflavones (11 mg daidzein and 4.38 mg genistein/capsule) at 8.00 a.m. after the bladder was emptied (Phyto Soy^®^, Laboratoires Arkopharma S.A., Carros, France). A first sample of urines (from 08.00 a.m. to 08.00 p.m.) was collected in a 2500 mL container containing 0.3 grams of boric acid and 0.2 grams of sodium ascorbate and stored in a refrigerator at 4 °C. A similar procedure was followed for a second sample of urines (from 08.00 p.m. to 08.00 a.m. of the following day). For each of urine sample the diuresis was determined and 50.00 mL was transferred to a falcon tube of 10 mL and stored at −20 °C until analysis. The data obtained were added and expressed as mg/24 hrs.

#### 2.3.6. Extraction of Urinary Phytoestrogens

Frozen urine rates were brought to room temperature (RT), vortexed and centrifuged at 1500× *g* for 20 min. Subsequently, 20.00 mL of supernatants were taken and mixed with 5.0 mL of 0.2 M acetate buffer (pH 4.0) and 100 μL of 10 mM flavone as internal standard. The mixture thus formed was loaded onto column C18 SPE reverse phase pretreated with 3 mL of methanol and 3 mL of acetate buffer. Upon completion of the urine passage through the column, the latter was washed with 2 mL of acetate buffer and the phytoestrogens were eluted with methanol and harvested in a falcon until an eluate volume of 2.00 mL was obtained. From this eluate were taken 100 μL and stored at −20 °C until HPLC analysis of non-conjugated phytoestrogens.

#### 2.3.7. Enzyme Hydrolysis of Urine Phytoestrogens

The remainder of the eluate (1.9 mL) was drained under nitrogen stream and incubated at 37 °C for 24 h after mixing with 0.9 mL of a solution containing 10 mL of 0.2 M acetate buffer (pH 4.0), 150 mg ascorbic acid and 500 μL glucuronidase/sulphatase (isolated from *Helix pomatia*). Hydrolyzed samples (total non-conjugated phytoestrogens and hydrolyzed conjugates) were then mixed with 1.0 mL of methanol and stored at −20 °C. Once taken to RT, the samples were vortexed and centrifuged at 850× *g* for 5 min and the supernatants used for HPLC analysis.

#### 2.3.8. Identification of Urinary Phytoestrogens

Different chromatographic runs were carried out on different biological matrices (urine and/or urine extracts) to identify the different phytoestrogens. At first, urine was analyzed as they came to the lab after the intake of the integrator. The urine was centrifuged at 1500× *g* for 20 min and the supernatant was filtered with 0.25 μM MILLEX-GP syringe filters. We adopted several experimental conditions with the aim of making the different phytoestrogens more visible and evaluable. For this purpose, we subjected solid phase extraction liquids using a resin coated HyperSep C18 column. Thus, the phytoestrogens present in the sample are retained by the resin and subsequently eluted and concentrated at a lower volume compared the starting volume, thus obtaining preconcentration of the analyte. Part of the eluate (0.1 mL) was analyzed by HPLC, the remaining volume subjected to enzymatic hydrolysis before analysis with glucoronidase/arylsulfatase, a procedure which allows us to well identify and evaluate the different phytoestrogens.

### 2.4. Statistical Analysis 

The results obtained are expressed as mean ± SD and statistical comparison between the two groups was performed using the Student’s t-test. Statistical significance was ascribed to the data when *p* < 0.05. If not specifically reported, each datum is representative of at least three different and separated experiments. The analyses were performed using SPSS software, version 23.0 (SPSS Inc., Chicago, IL, USA).

## 3. Results

Seventeen different species were identified, 10 of which were Gram-negative anaerobes bacteria, all belonging to the *Bacteroides* genus. 

Table 2 reports the different, most abundant, bacterial species identified in the fecal samples of the forty subjects participating to the study. 

**Figure 1:**
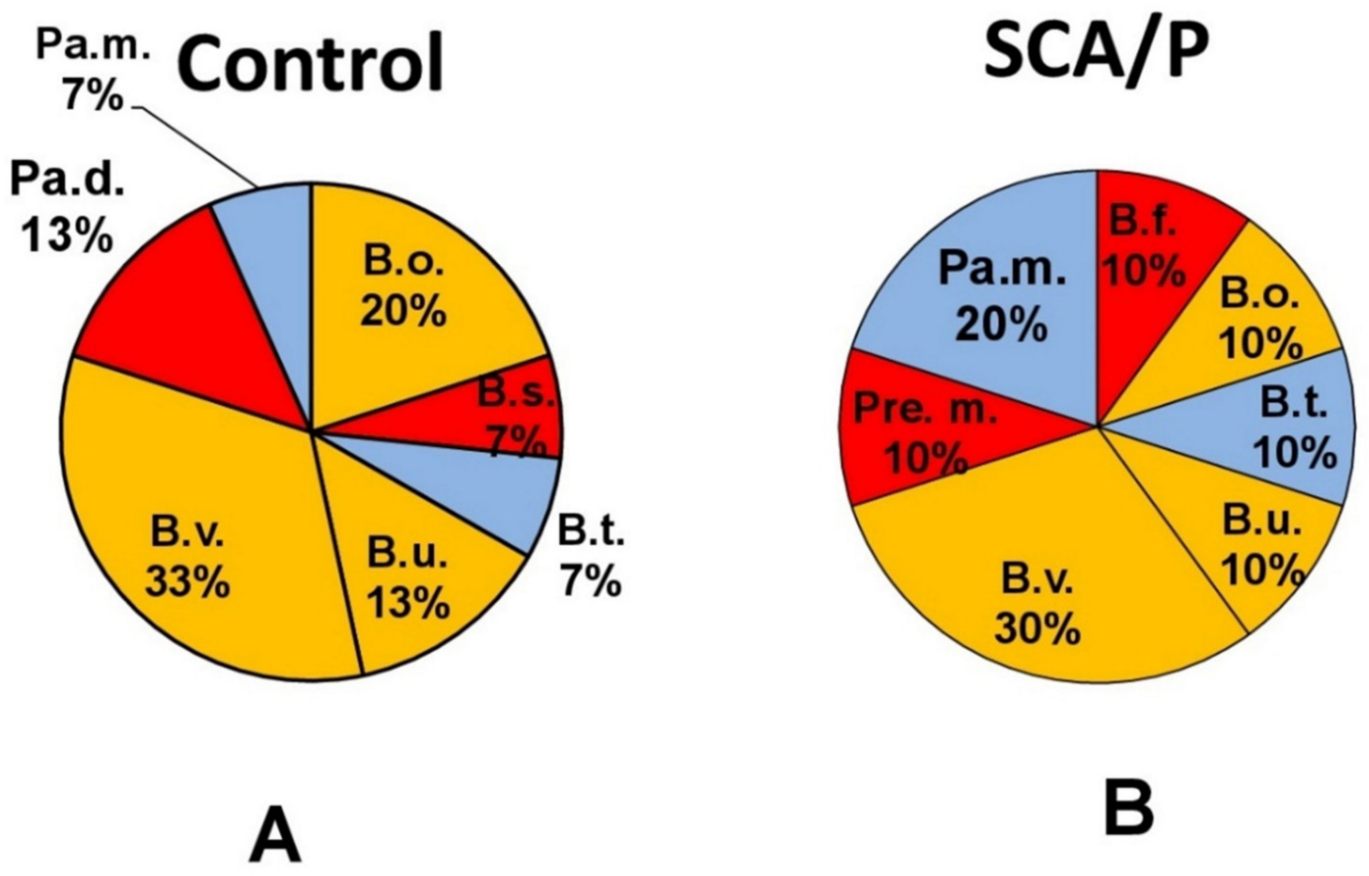
Prevalence of bacterial species in control subjects (**A**) and SCA/P patients (**B**)**.**

Figure 1A,B reports the bacterial species, respectively, identified in control or in SCA/P fecal samples. A significant difference of bacteria species, specifically in the *Bacteroides* group, was evidenced between the two groups of subjects. The presence of *Parabacteroides distasonis (Pa.d.)*, *Clostridium clostridioforme* (*C.c.*) and *Pediococcus pentasaceus* (*Pe.p.*) was detected only in “control” fecal samples. However, only in SCA/P fecal samples, *Bacteroides fragilis* (*B.f.*) and *Prevotella melaninigenica* (*Pre.m.*) were identified. In addition, a greater presence of *Bacteroides ovatus (B.o.*), *Bacteroides uniformis* (*B.u.*) and *Bacteroides vulgatus* (*B.v.*) (20%, 13% and 33% respectively) were detected in control fecal samples compared to SCA/P subjects fecal samples (10%, 10% and 30% respectively). 

**Figure 2:**
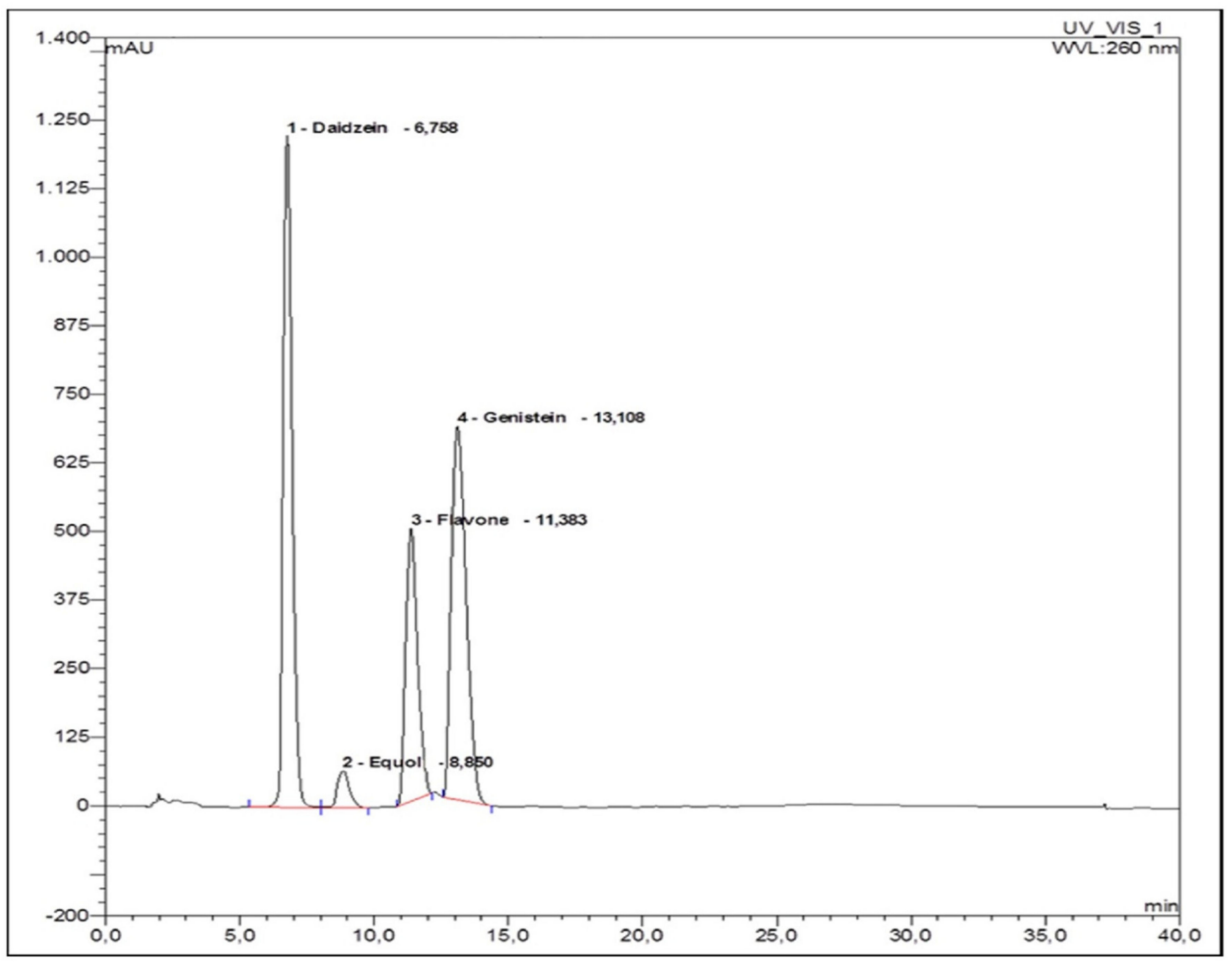
High performance liquid chromatographic (HPLC) chromatogram of 0.5 mM soy derivatives.

Figure 2 shows a HPLC chromatogram obtained running a standard solution containing daidzein, equol, flavone and genistein at a 0.5 mM concentration each. The retention times (min) recorded for the 4 molecules were: 6.75, 8.85, 11.38 and 13.10 min respectively. 

Once established HPLC conditions able to identify soy derivatives (Figure 2), in order to quantify the urinary amount of daidzein, genistein and equol, three different calibration curves were realized (Supplementary Figure S1), as reported in Materials and Methods. 

**Figure 3:**
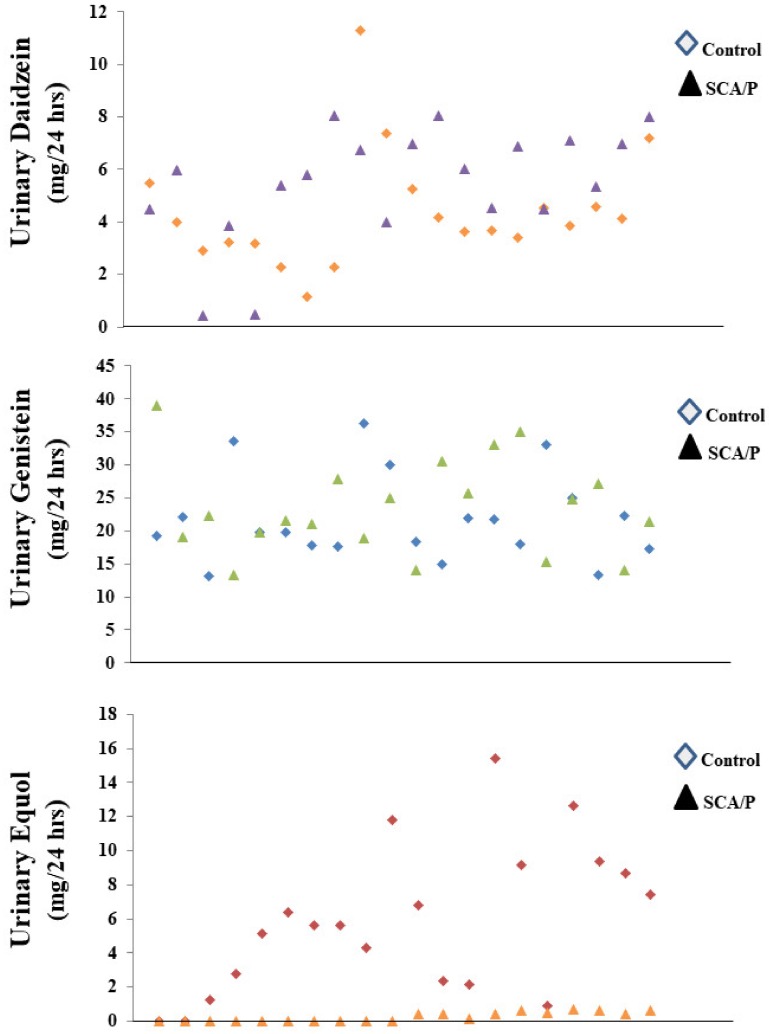
Daidzein, genistein and equol levels, detected by HPLC, in urine samples of controls and SCA/P subjects.

The levels of urinary soy derivatives are reported in Figure 3.

**Figure 4:**
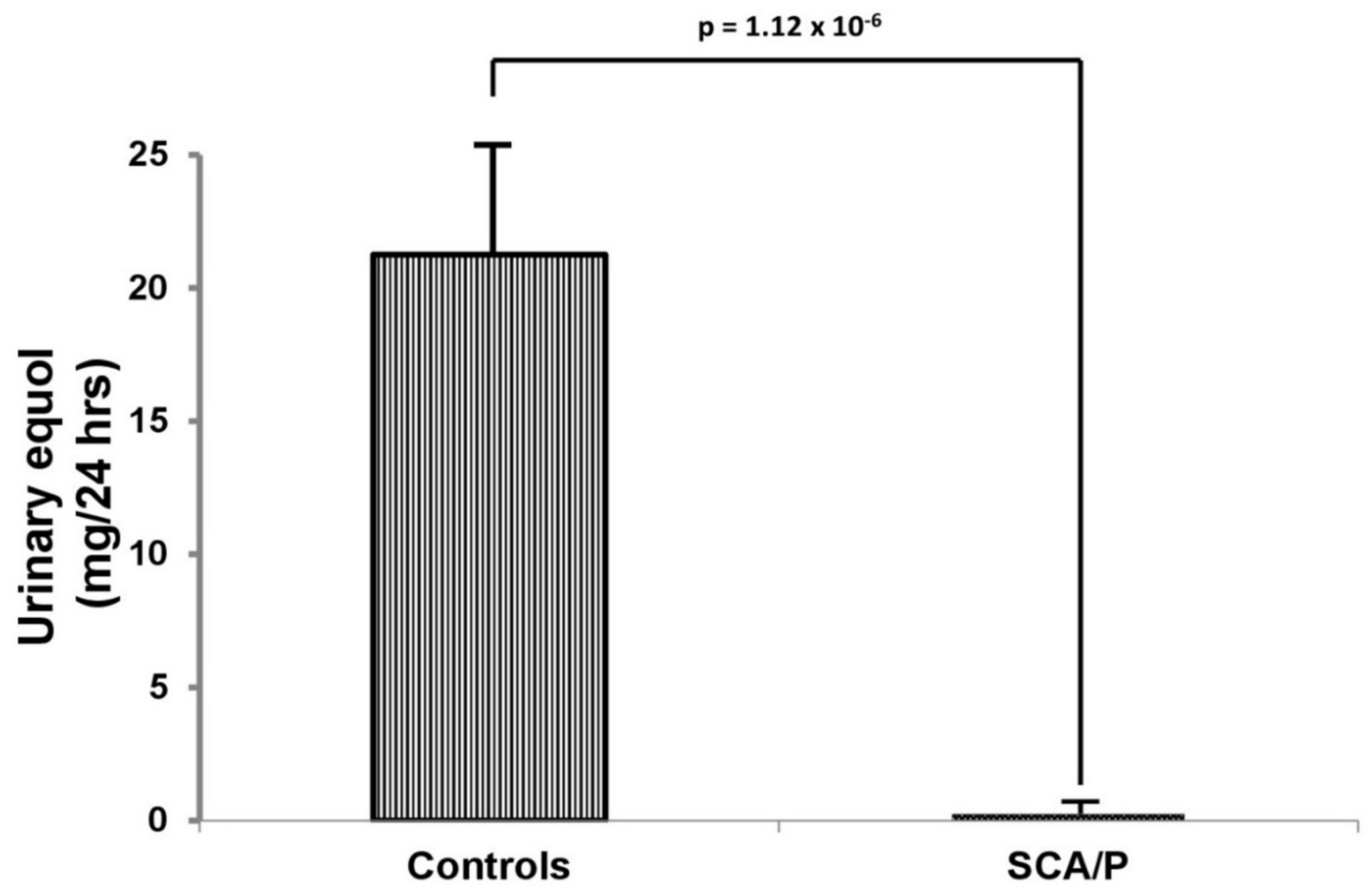
Urinary concentrations of equol in control and SCA/P subjects.

No statistically significant differences were revealed in genistein (4.9 ± 2.2 mg/24 hrs and 5.5 ± 2.1 mg/24 hrs, respectively, *p* = 0.12) and daidzein (21.7 ± 6.6 mg/24 hrs and 23.5 ± 7.2 mg/24 hrs, respectively; *p* = 0.44) urinary levels between the two groups of subjects. On the contrary, the urinary equol levels were different in the two groups of subjects. In particular, equol was undetectable in the control subjects only in two subjects, while it was undetectable in ten of the twenty SCA/P subjects, determining a statistically significant difference between control and SCA/P subjects (21.25 ± 4.3 mg/24 hrs and 0.24 ± 0.27 mg/24 hrs, respectively, *p* = 1.12 × 10^−6^), as reported in Figure 4.

**Figure 5:**
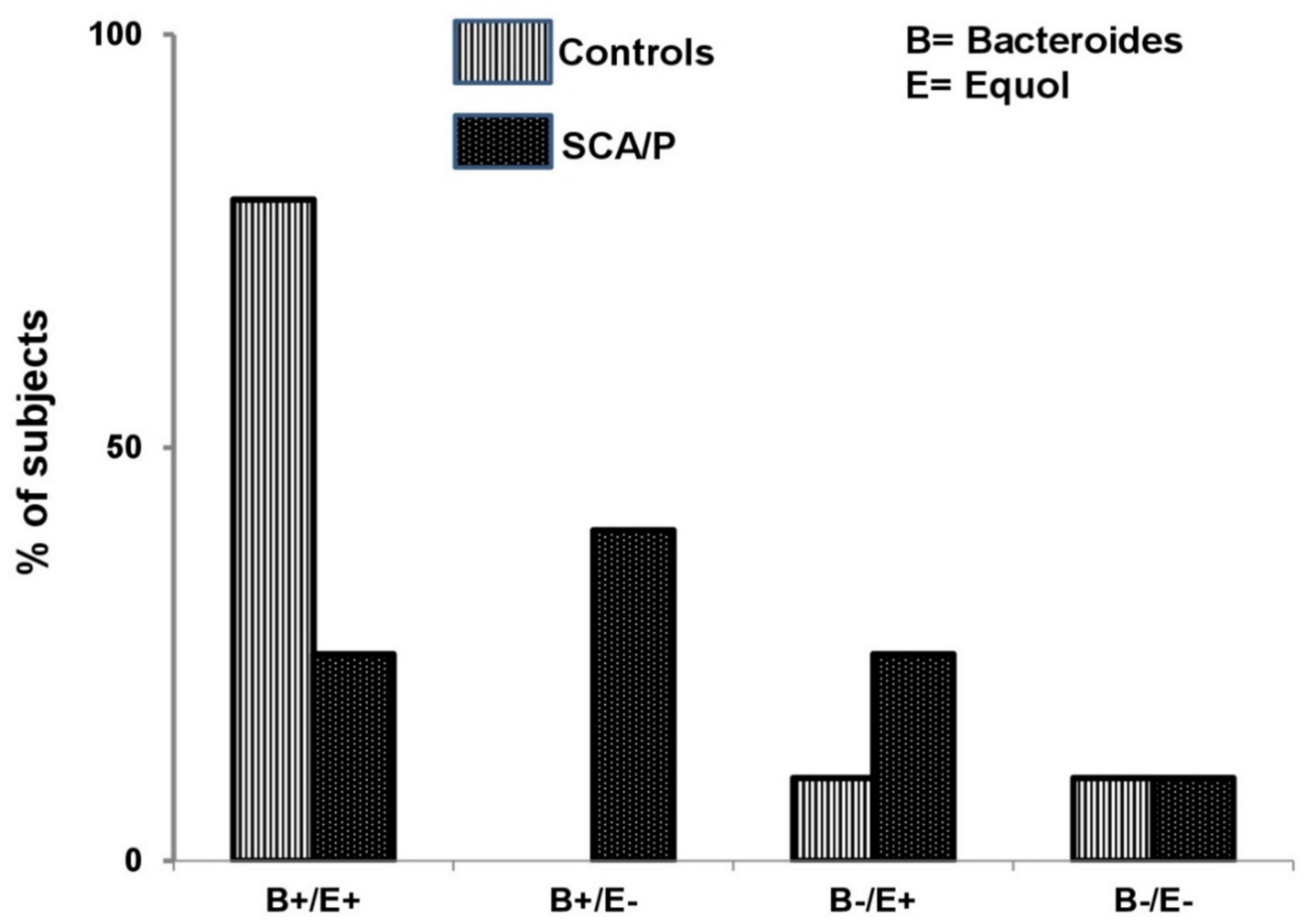
Fecal *Bacteroides* strains and urinary equol presence.

In Figure 5 the percent of control and SCA/P subjects reporting, at the same time, the presence or absence of urinary equol (E+/E-) and the presence or absence of fecal *Bacteroides* (B+/B-) is reported. In detail, the 80% of subjects of control group showed urinary equol and fecal *Bacteroides* (B+/E+), while only in the 25% of SCA/P patients a similar combination was evidenced. Ten percent of the control subjects resulted negative for fecal *Bacteroides* and positive for urinary equol (B-/E+), and 10% both negative for fecal presence of *Bacteroides* and for urinary equol (B-/E-). No subjects, in the control group, reported the presence of fecal *Bacteroides* in the absence of urinary equol (B+/E-). In the SCA/P group, 25% of subjects revealed the presence of urinary equol in the absence of fecal *Bacteroides* (B-/E+), 10% resulted negative for both urinary equol and fecal *Bacteroides* (B-E-) and 40% were positive for fecal *Bacteroides* and negative for urinary equol (B+/E-).

## 4. Discussion

In the present study, soy metabolism and gut anaerobic microbiota were evaluated in subjects with sporadic colorectal adenomas (SCA/P group) and compared to similar evaluations obtained from subjects without proliferative lesions (control group). The data clearly indicate that soy metabolism and gut microbiota of SCA/P subjects are different from those of controls subjects. As detailed in Figure 3, no significant differences were evidenced for daidzein and genistein urinary excretion, while urinary equol levels was undetectable in the 50% (10/20) of SCA/P patients and in the 10% (2/20) of control group subjects. In addition, SCA/P patients presenting detectable equol urinary excretion (50%) had a statistically significant lower equol levels compared to controls (90%) (Figure 4). These results, together with the data from literature, reporting a significantly lower occurrence for CRC in subjects who are consumers of a naturally rich soy diet and, presumably, that has greater availability of soy metabolites [17,18], suggesting that control group subjects could have a milieu able to contrast the development of the carcinogenetic process. This could be due to the presence, in their gut microbiota, of a genetic determinant able to metabolize daidzein in equol, a well-known antioxidant and anti-carcinogenic molecule [29]. The greater presence of equol in the urine samples of control subjects, compared to SCA/P patients, seems to be associated to a variability of gut microbiota and in particular to a specific presence of *Bacteroides* species, anaerobic bacteria reported able to metabolize daidzein in equol [34]. According to this hypothesis, in the present study, 80% of control subjects and 25% of SCA/P patients reported, at the same time, detectable equol urinary levels and the presence of *Bacteroides* species in their feces (Figure 5). Equol is the molecule which exerts the most significant estrogenic effects among soy isoflavones, shows a potent antioxidant activity, is produced only by the intervention of human gut microbiota [35] and is synthetized only by approximately 30%–40% of the adult population following a soy-based diet. 

Interindividual variation in the production of equol from daidzein is attributed, in part, to the differences in the diet and in gut microbiota composition. Cai et al. [36] have demonstrated that equol inhibits cell proliferation in colon cancer cell line. Tumor formation in the colon begins with the transition of a normal epithelium to a state of hyperplasia due to an increased cell proliferation. Consequently, epithelial architecture loses its characteristic shape and organization and becomes dysplastic [4,37]. This dysplasia has the potential to develop into a nonmalignant adenoma, as mostly revealed in our SCA/P subjects. 

Concerning the role of gut microbiota in colon carcinogenesis, many authors have found a decreased microbial diversity in CRC tissue samples compared to nearby normal colonic tissues. These changes were associated with a reduction of certain bacterial genera like *Clostridium* and *Bacteroides* [38,39]. The stability of the intestinal microorganism community and the diversity of microbial species both define a healthy gut microbiota in adulthood. More specifically, despite lifestyle and food changes, the *Firmicutes* (such as *Lactobacillus*) and the *Bacteroides* represent the main bacterial phyla in the gut [40], constituting an ecological community entertaining a beneficial relationship with the host [41]. It is widely accepted that colon-producing equol microorganisms are anaerobic bacteria [42,43] and previous experimental studies have allowed to hypothesize that the *Bacteroides* genus may play an important role in equol production [43]. 

An imbalance of the intestinal bacteria composition (dysbiosis) could lead to different diseases. However, during the carcinogenetic evolution, the shift of microbial community composition may be conversely due to the inhospitable tumor environment, characterized by several aspects: the presence of rapidly growing tumor cells competing for nutrients, the infiltrating immune cells producing inflammatory compounds ((reactive nitrogen species (RNS) and reactive oxygen species (ROS)), which can be toxic to microbes and exert genotoxic effects on the proliferating cells [44]. We believe that the importance of antioxidants in counteracting oxidative stress and preventing colon carcinogenesis highlights the potential protective role of antioxidants [36], while the absence and/or the reduced presence of an antioxidant agent, such as equol, and the equol producing microorganisms, could represent a predisposing carcinogenetic condition. The difference in the gut microbiota pattern of the two groups of subjects, particularly evident for the *Bacteroides* genus, can support this hypothesis. In fact, it is important to underline the presence of *Parabacteroides distasonis* (*Pa.d.*), which is known to exert anti-inflammatory and anti-cancer properties [25], only in stool samples of control subjects, as well as the presence, of the enterotoxigenic *Bacteroides fragilis* (*B.f.*) and *Prevotella melaninigenica* (*Pre.m.*) species, only in SCA/P stools samples, which are suspected to be associated with CRC development [45] stimulating exaggerated immune responses via Th17 cells [46], as for other gastrointestinal cancers and other pathologies [46,47,48,49,50,51]. Considering such patterns and literature data demonstrating the ability of natural products and probiotics to modulate the immune system [52,53,54,55,56], a lot of studies are currently aimed at evaluating the effectiveness of them for the prevention of intestinal cancer [57,58].

Our study presents some limitations: 1) the small number of subjects enrolled requires to extend our evaluations to a larger population; however, the striking differences observed between controls and SCA/P group strongly support our approach; 2) we evaluated only anaerobic intestinal bacteria, and in particular *Bacteroides* since they represent a large bacterial genus present in the gut with an important role in equol production; 3) the type of the study did not allow us to explore the condition at the beginning of the process leading to polyp-dysplasia formation, which requires a long time frame; however, this is a limitation of all human studies involving dietary habits and the related evolution of gut microbiota. The fact that about 40% of the general population do not process soy into equol and that 40% of the SCA/P group had *Bacteroides* but not equol detected (B+E-) and 25% of them had equol but not *Bacteroides* (B-E+) highlights the complexity of gut microbiota composition/interplay. 

## 5. Conclusions

In conclusion, the results obtained in this study suggest that the presence of *Bacteroides* genus in the fecal samples of control subjects seems to be involved in the daidzein transformation to equol, an antioxidative, anti-carcinogenic molecule, which shows the most active estrogenic properties of soy isoflavones. 

## Figures and Tables

**Table 1:** Main demographical and anthropometric features of the subjects enrolled in the study.

	SCA/P	Controls	*p*-Value
Age (yrs)	63.4 ± 8	64.5 ± 4	0.56
Sex ratio (M/F)	14:6	11:9	0.61
BMI	29.8 *±* 5.4	30.3 *±* 7.2	0.76
Weight (Kg)	80.5 *±* 12.3	77.3 *±* 13.8	0.45

**Table 2:** The main bacterial species from fecal samples in the study population.

Gram - bacteria	Gram + bacteria
1. *Bacteroides fragilis* (B.f.)	1. *Bifidobacterium* spp (Biff.)
2. *Bacteroides ovatus* (B.o.)	2. *Clostridium clostridioforme* (C.c.)
3. *Bacteroides stercoris* (B.s.)	3. *Clostridium innocuum* (C.i.)
4. *Bacteroides thetaiotaomicron* (B.t.)	4. *Clostridium ramosum* (C.r.)
5. *Bacteroides uniformis* (B.u.)	5. *Clostridium sardelli* (C.s.)
6. *Bacteroides vulgatus* (B.v.)	6. *Corynebacterium xerosus* (Cor.x.)
7. *Prevotella melaninigenica* (Pre.m.)	7. *Propionibacterium avidum* (Pr.a.)
8. *Parabacteroides distasonis* (Pa.d.)	
9. *Parabacteroides merdae* (Pa.m.)	
10. *Pediococcus pentasaceus* (Pe.p.)	

## Supplementary Materials

The following are available online at https://www.mdpi.com/2076-2607/8/4/469/s1.
