# Supplementary figures and images for: Soy Metabolism by Gut Microbiota from Patients with Precancerous Intestinal Lesions
Source: Microorganisms. 2020 Mar 25;8(4):469. doi: 10.3390/microorganisms8040469 (PMC7232402; doi:10.3390/microorganisms8040469)

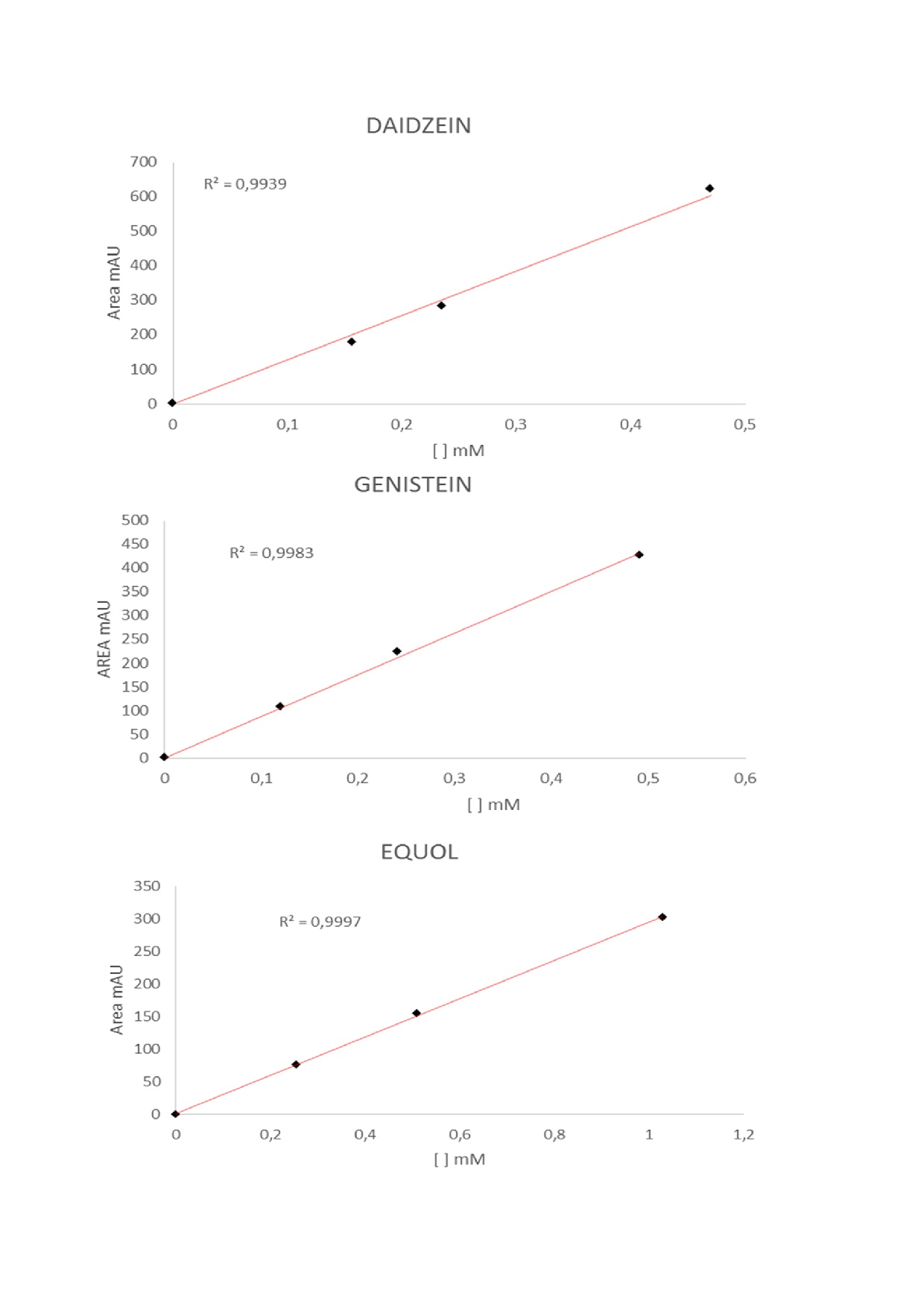

Supplement: Supplementary file 1 [file microorganisms-08-00469-s001.jpg]
